# Two Cases of Satisfactory False Lumen Thrombosis Achieved via Total Arch Replacement Using the Frozen Elephant Trunk Technique for Chronic Aortic Dissection

**DOI:** 10.7759/cureus.95104

**Published:** 2025-10-21

**Authors:** Daigo Shinoda, Kosuke Miyoshi, Nobu Yokoyama, Manabu Shiraishi

**Affiliations:** 1 Department of Cardiovascular Surgery, Tokyo Metropolitan Bokutoh Hospital, Tokyo, JPN

**Keywords:** chronic aortic dissection, false lumen, frozen elephant trunk, thoracic endovascular aortic repair, total arch replacement

## Abstract

We report two cases of chronic aortic dissection (CAD) that were successfully treated with total arch replacement (TAR) using frozen elephant trunk (FET). Case 1 was a 64-year-old female with incidentally detected CAD. Surgery was planned due to progressive aortic enlargement. The primary entry was in the distal arch, and TAR with FET was performed. Case 2 was a 53-year-old male with an abnormal chest radiography, later diagnosed as CAD. He also underwent TAR with FET. Both patients were discharged without complications. Postoperative computed tomography at six months showed true lumen expansion and favorable false lumen thrombosis.

## Introduction

Various treatment options are available for cases of chronic aortic dissection (CAD) with the distal aortal arch as the primary entry. These include descending aortic replacement via left thoracotomy, thoracic endovascular aortic repair (TEVAR), and hybrid approaches that combine open surgery with endovascular techniques. Each of these strategies has its advantages and disadvantages. In cases of aortic dissection, the frozen elephant trunk (FET) technique has been reported to be helpful as an adjunct to total arch replacement (TAR), as it facilitates decompression of the false lumen (FL) in the distal aorta and promotes thrombosis formation [1,2]. The FET technique was first reported by Karck et al. in 2003 [3]. The Japanese-made J Graft FROZENIX FET prosthesis (Japan Lifeline Inc., Tokyo, Japan) was introduced in 2014 [4,5]. Few reports in the literature have described the morphological changes that take place in cases of aortic dissection (e.g., FL thrombosis) following treatments aimed at closing the primary CAD entry. Herein, we present two cases in which favorable FL thrombosis was achieved following TAR via the FET technique.

This study was presented in part at the 53rd Annual Meeting of the Japanese Society for Vascular Surgery, held in Kitakyushu, Japan, in May 2025.

## Case presentation

Case 1

A 64-year-old female was incidentally diagnosed with CAD and subsequently underwent regular computed tomography (CT) imaging. Surgical intervention was planned, as progressive aortic enlargement was observed. The primary CAD entry was identified in the distal aortic arch. TAR was performed using a 23 mm FROZENIX Partial ET graft (Japan Lifeline Inc.), resulting in the successful closure of the primary entry.

The patient was extubated on postoperative day (POD) one and transferred from the intensive care unit to the general ward on POD seven. Delayed-phase contrast-enhanced CT performed postoperatively confirmed full closure of the primary entry. Although there was no reduction in the diameter of the FL, favorable FL thrombosis was observed. The patient was discharged on POD 28 without complications.

**Figure 1:**
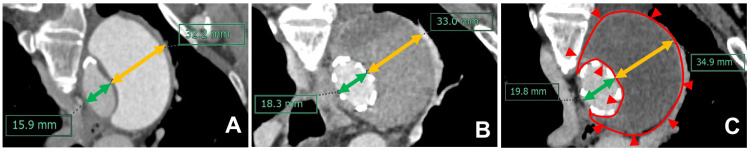
Serial contrast-enhanced computed tomography (CT) images of case 1. A: Preoperative CT. The true lumen diameter was 15.9 mm (green line), while that of the false lumen was 32.2 mm (yellow line). B: CT before the patient’s discharge from the hospital. The true lumen diameter increased slightly to 18.3 mm (green line), while that of the false lumen increased to 33.0 mm (yellow line). Although no reduction in false lumen size was observed, favorable thrombosis was achieved. C: CT performed at six months postoperatively. The true lumen diameter had further increased to 19.8 mm (green line), while that of the false lumen measured 34.9 mm (yellow line). The false lumen remained well thrombosed (the red outlined area), with continued expansion of the true lumen.

Six months postoperatively, follow-up contrast-enhanced CT showed no significant change in the aneurysm’s size; however, 24% expansion of the true lumen was noted. The FL remained well-thrombosed, and the patient’s postoperative course was otherwise uneventful (Figure 1).

Case 2

A 53-year-old male was referred to our department after an abnormal shadow was detected on a chest radiograph. Further evaluation led to a diagnosis of CAD. TAR was performed using a 29 mm FROZENIX Partial ET graft (Japan Lifeline Inc.), resulting in the successful closure of the primary entry.

The patient developed pneumonia postoperatively, which was managed conservatively. He was extubated on POD seven and transferred from the intensive care unit to the general ward on POD 10. Delayed-phase contrast-enhanced CT confirmed successful closure of the primary entry and evidence of FL thrombosis.

**Figure 2:**
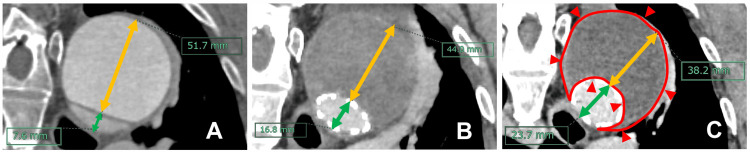
Serial contrast-enhanced computed tomography (CT) images of case 2. A: Preoperative CT. The true lumen diameter was 7.6 mm (green line), while that of the false lumen was 51.7 mm (yellow line). B: CT before the patient’s discharge from the hospital. The true lumen had expanded to 16.8 mm (green line), while that of the false lumen had decreased to 44.0 mm (yellow line). Favorable false lumen thrombosis was observed, accompanied by false lumen shrinkage and true lumen expansion. C: CT performed at six months postoperatively. The true lumen diameter had further increased to 23.7 mm (green line), while that of the false lumen had shrunk to 38.2 mm (yellow line). The false lumen remained well-thrombosed (the red outlined area), with continued false lumen shrinkage and true lumen enlargement.

The patient was discharged on POD 22 without complications. Six months postoperatively, follow-up CT revealed a 26% reduction in FL thickness, expansion of the true lumen, and shrinkage of the FL. The FL remained well-thrombosed, and the patient’s postoperative course was otherwise uneventful (Figure 2).

Operative technique

The procedure was performed via median sternotomy. After a standard dose of heparin was administered, cardiopulmonary bypass was established through cannulation of the right femoral artery, left axillary artery, superior vena cava, inferior vena cava, and left ventricular vent. Circulatory arrest under moderate hypothermia (bladder temperature, 25°C) was initiated, and the ascending aorta was opened to establish selective antegrade cerebral perfusion.

**Figure 3:**
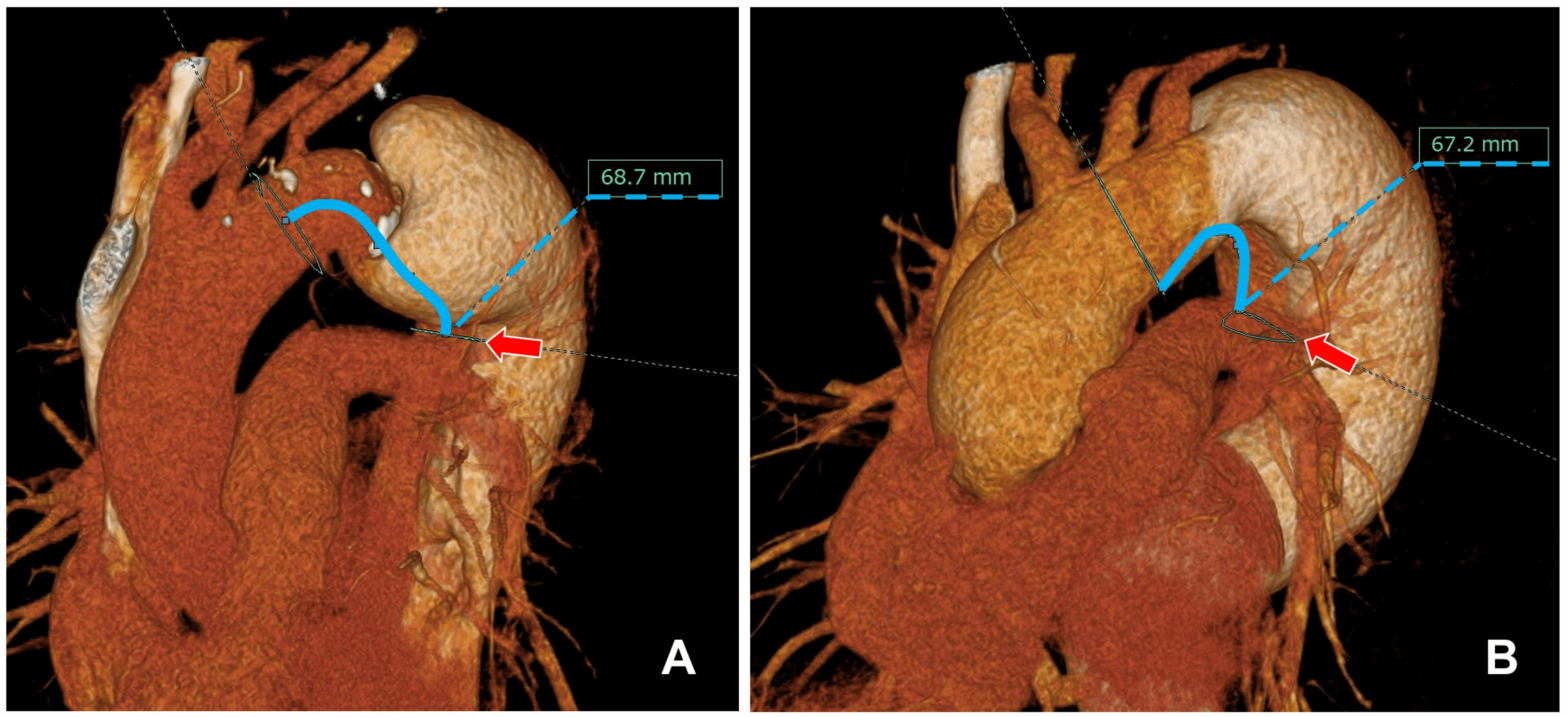
Preoperative simulation using contrast-enhanced computed tomography with multiplanar reconstruction. A: Case 1. B: Case 2. The planned distal end of the frozen elephant trunk (FET) was positioned to ensure an appropriate landing zone (arrows), and the distal anastomosis site of the vascular graft was determined accordingly, taking into account the selected length of the FET (blue line).

When using the J Graft FROZENIX device (Japan Lifeline Inc.), preoperative contrast-enhanced CT with multiplanar reconstruction was used to measure the distance from the distal anastomotic site of the four-branched graft to the intended distal end of the FET (Figure 3). The FET length was selected to avoid the curvature of the proximal descending thoracic aorta and ensure deployment above the level of the eighth thoracic vertebra. Based on this preoperative imaging, the optimal FET diameter was determined to be 90% of the maximum actual lumen diameter of the descending aorta. In case 1, the maximum true lumen diameter was 27 mm; hence, a 23 mm FET was selected. In case 2, where the maximum true lumen diameter was 33 mm, a 29 mm FET was used.

**Figure 4:**
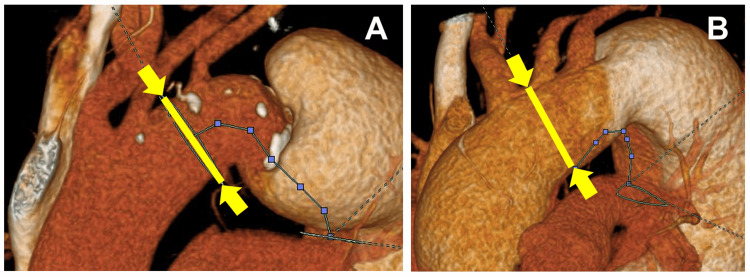
Site of aortic transection. A: In case 1, the aorta was transected between the left common carotid artery and the left subclavian artery (yellow line). B: In case 2, the aorta was transected between the brachiocephalic artery and the left common carotid artery (yellow line).

The site of aortic transection for each case was determined based on preoperative imaging: between the left common carotid and left subclavian arteries for case 1 and between the brachiocephalic and left common carotid arteries in case 2 (Figure 4). The FET was inserted into the true lumen of the descending aorta using a guidewire. After confirming the distal FET’s position via transesophageal echocardiography, it was deployed, and distal anastomosis was performed using circumferential pledgeted sutures. The proximal anastomosis was ultimately completed using more circumferential pledgeted sutures after systemic circulation was resumed. Once the aortic cross-clamp was removed, the branch grafts were sequentially anastomosed end-to-end to the left subclavian, left common carotid, and brachiocephalic arteries.

Blood transfusion management was conducted in accordance with standard institutional transfusion protocols. Preoperative cerebrospinal fluid drainage was not performed. No patients experienced paraparalysis or permanent surgical deficits due to spinal cord ischemia during the postoperative period.

## Discussion

According to the latest annual report on thoracic and cardiovascular surgery in Japan, the in-hospital mortality rate for descending aortic replacement in patients with chronic type B aortic dissection is 4.5%, increasing to 11.0% for thoracoabdominal aortic replacement [6]. As such, one-stage open surgical graft replacement via left thoracotomy is highly invasive, and the operative mortality for CAD, therefore, remains significant. In recent years, TEVAR has increasingly been considered a safer alternative for treating various aortic pathologies, including CAD. Conway et al. reported favorable outcomes using TEVAR for CAD, with a mortality rate of 2.4%, stroke rate of 0.8%, and paraplegia rate of 2.4% [7]. However, owing to anatomical limitations, TEVAR may not be feasible in certain cases. Since TEVAR requires an adequate landing zone in a healthy segment of the aorta, the presence of aneurysmal or dissected changes in the aortic arch increases the complexity of this procedure. Although alternative strategies, such as TEVAR with branch reconstruction or fenestrated TEVAR, are available, postoperative patient management can become particularly challenging if aneurysmal enlargement occurs in the distal aortic arch near the primary entry site following these procedures.

By contrast, the use of the FET technique via median sternotomy during TAR, as demonstrated by the present two cases, offers several significant advantages. The absence of major neurological complications such as stroke or persistent paraparalysis in both of our cases supports the view that this approach has an acceptable risk profile. Most notably, it reduces surgical invasiveness compared with left thoracotomy and provides closure of the primary entry equivalent to that achieved via TEVAR. An additional theoretical benefit of this approach is the elimination of the risk of type Ia endoleaks, which can occur following TEVAR. Therefore, if further treatment is required for late aneurysmal enlargement, the previously implanted FET can serve as a reliable distal landing zone for TEVAR or a proximal anastomotic site for future open repairs, thereby facilitating secondary interventions.

Ryomoto et al. reported postoperative aorta-related event rates of 19% following open thoracotomy and 45% following TEVAR [8], highlighting the superiority of open thoracotomy in terms of reducing postoperative aorta-related events. Ito et al. reported a late aorta-related event rate of 44.2% when performing TAR using the FET technique via median sternotomy [9], which is comparable to what has been reported for TEVAR. However, open thoracotomy imposes a greater physiological burden in terms of surgical invasiveness than TEVAR or TAR using the FET technique, potentially accelerating patient deconditioning and posing a significant barrier to postoperative functional recovery and return to daily life. Therefore, the indications for open thoracotomy should be carefully considered, particularly in patients with frailty.

Regarding surgical invasiveness, the median sternotomy approach offers significant advantages over left thoracotomy. Although the potential for additional mid- to long-term aorta-related interventions remains similar to that of TEVAR, TAR using FET can be performed even in anatomically challenging cases where TEVAR is not feasible. Furthermore, it provides the added benefit of facilitating secondary interventions during later phases, owing to the presence of a well-defined distal landing zone or proximal anastomotic site established by the FET.

Dohle et al. reported on postoperative aortic remodeling following the FET procedure in patients with aortic dissection [1]. In their study, changes in the luminal volume in the distal aorta were measured to assess the remodeling process. Volumetric measurements were performed across the entire descending and abdominal aortal sections, which were divided into three segments. Segment A extended from the distal anastomosis site of the FET to the end of the stent graft; segment B extended from the distal end of the stent graft to the origin of the celiac artery; and segment C extended from the celiac artery to the aortic bifurcation.

Postoperative CT revealed complete FL thrombosis in segment A in 82% of their patients with CAD. This rate remained unchanged at 82% at one year postoperatively, with 14% of the cases demonstrating complete obliteration of the FL. In segment B, the rate of FL thrombosis increased from 23% immediately postoperatively to 41% one year later. Although no cases of complete FL thrombosis were observed in segment C, CT performed regularly over the first year postoperatively showed progressive enlargement of the true lumen along the stent graft, along with reductions in both the FL and overall aortic diameter [1]. Both cases corresponded to segment A as defined by Dohle et al. in the present study, and favorable FL thrombosis was observed postoperatively in both patients. According to Dohle’s findings, these observations suggest promising prognoses for sustained FL thrombosis and potential future reductions in FL volume.

FROZENIX is a commercially available FET prosthesis offered in three variants in Japan: Conventional, Partial ET, and FROZENIX 4 Branched, each with distinct characteristics. We used the FROZENIX Partial ET for our present two cases. This graft is designed with thinner wires in the stent structure than the conventional version, thus reducing both the spring-back and radial expansion forces. It also features a 20 mm skirt at the distal end to facilitate future anastomoses in potential descending aortic replacement procedures.

Some reports have suggested that a staged surgical approach using the FROZENIX Partial ET may be effective for managing complex aortic pathologies, particularly in high-risk patients [10]. These findings underscore the importance of developing long-term treatment strategies for managing complex CAD cases. Although the FET technique offers significant therapeutic potential, it is not without limitations. One notable concern is the development of distal stent graft-induced new entry, which can both impede FL thrombosis and necessitate early re-intervention. Therefore, careful consideration of FET sizing and spring-back force is essential.

At our institution, we routinely select FROZENIX Partial ETs with diameters of ≤90% of the descending aorta’s maximum true lumen diameter when treating cases of CAD. The FROZENIX Partial ET has a fixed stent skeleton length of 60 mm. Accordingly, thorough preoperative assessments are performed using multiplanar reconstructed CT imaging to evaluate each patient’s aortic morphology and primary entry location. This facilitates precise planning of the distal anastomotic site and determination of the required length of the non-stented graft portion beyond the stent’s skeleton.

Although the present report discusses very short-term postoperative observations, we have recently encountered two cases in which the use of the FET technique produced effective FL thrombosis. Long-term follow-up will be conducted, with the expectation of observing further reductions in aortic diameters and FLs. The continued accumulation of clinical experience related to this promising approach is warranted.

## Conclusions

Proper execution of the FET technique resulted in favorable FL thrombosis and yielded satisfactory short-term clinical outcomes in two cases of CAD that we recently treated. Given the potential for subsequent reductions in aortic diameters and FLs with this approach, continued follow-up of these two cases and the further accumulation of additional cases from other clinical settings are warranted.
